# Fabrication of Environmental-Friendly Magnetite Nanoparticle Surface Coatings for the Efficient Collection of Oil Spill

**DOI:** 10.3390/nano11113081

**Published:** 2021-11-15

**Authors:** Mahmood M. S. Abdullah, Hamad A. Al-Lohedan

**Affiliations:** Department of Chemistry, College of Science, King Saud University, P.O. Box 2455, Riyadh 11451, Saudi Arabia; hlohedan@ksu.edu.sa

**Keywords:** oil spill collectors, magnetite nanoparticles, sodium alginate

## Abstract

Over the past few decades, there has been an increased trend for the use of natural compounds and their derivatives as alternatives to traditional chemicals and is due to their renewability, green character, and wide availability. This work aims to convert sodium alginate (S.ALG), a natural polysaccharide, into amides through its conversion to alginic acid (H.ALG). The formed H.ALG was esterified using methanol, followed by a reaction with octadecylamine (OA) and dodecylamine (DA) to produce corresponding amides, OA-ALG, and DA-ALG, respectively. The synthesized OA-ALG and DA-ALG were used as capping agents to further form hydrophobic magnetite nanoparticles (MNPs), OA-MNPs and DA-MNPs, respectively. The chemical structures, morphology, hydrophobicity, and magnetic properties of OA-MNPs and DA-MNPs were investigated using different instrumental techniques. Furthermore, the efficacy of as-synthesized MNPs as oil spill collectors were also evaluated using different ratios of MNPs:crude oil. From the analysis of results, the OA-MNPs and DA-MNPs exhibited high efficiency in the collection of oil spill even at low ratios of MNPs:crude oil.

## 1. Introduction

Oil spills in the oceans due to ship accidents, tanker collisions, and platforms represent one of the most common causes of marine pollution, where such spills serve as a basis for the immediate and long-term damage to aquatic and coastal regions [1,2]. There have been many different techniques that can be applied to control these disasters including the physical, chemical, and bioremediation [3,4]. Among them, the chemical technique is one of the most applied techniques due to its low cost, fast treatment, and low effort. Commonly, chemicals are used for dispersing, sorbing or collecting oil spill [4]. Oil spill dispersants are chemicals sprayed on the crude oil slick to reduce oil/water interfacial tension leading to break-up of the oil into small drops that are easily biodegraded with time. However, these dispersants may also have negative effects on the environment and contribute significant damage to aquatic and marine life [5]. The oil sorbers are chemicals which have a high uptake for oil spills over water, although they have limited reusability especially for highly viscus crude oils [6]. The usage of oil spill collectors is considered one of the efficient means of accumulating the spilled crude oil, in addition to their reusability for the high viscus heavy crude oils with minimal damage to marine life [7,8].

Over the past few years, factionalized magnetite nanoparticles (MNPs) have attracted considerable attention due to their high adsorption capacity, magnetic properties, and high surface area. They have been applied in different fields, for example, water treatments [9,10], oil spill remediation [7,11], and biomedical applications [12]. The performance of MNPs in these applications is essentially affected by their surface functionalization. Several chemicals have been reported to functionalize the MNPs’ surfaces to improve their dispersibility, chemical stability, and efficacy in desired applications. With increasing global awareness for the usage of low toxic materials, researchers are focusing on the incorporation of natural compounds as alternatives to conventional chemicals, which are usually associated with high toxic levels and low efficiency. In oil spill remediation, several studies have reported the usage of plants including cotton, kapok, rice straw banana skins, potato peel and so forth, to combat this type of disaster [13,14]. In addition, natural compounds, such as asphaltenes [6] and plant extracts [4,15,16], have been applied for functionalization of the surface of MNPs and for the collection of oil spills. The use of natural compounds for oil spill remediation eliminates the negative effects of conventional chemicals that in general serve as secondary sources of marine environmental pollution [4]. Another study reported the use of modified polyelectrolyte-MNPs for functionalization of the cell walls of the bacteria *Alcanivorax borkumensis,* enhancing the efficacy of this bacteria for oil spill biodegradation [17]. Similarly, sodium alginate (S.ALG) is a natural polysaccharide compound extracted from brown algae and has been used in cosmetic, food and several different biomedical applications. In the food and cosmetics industries, it has been used as a thickening or viscosity increasing agent [18], while in the biomedical sector it has been used as a natural agent for enhancing wound healing and tissue generation capacity [19]. Therefore, keeping in view the advantages of using naturally available compounds as oil spill collectors, our earlier studies deal with the usage of plant extracts and asphaltenes for the preparation of magnetite nanoparticles (MNPs) for the collection of oil spilled [4,6,16]. Herein, we report the use of S.ALG for the preparation of renewable amides; such formed amides were used as capping agents for magnetite nanoparticles (MNPs). Following the synthesis, the chemical and physical properties, such as chemical structures, crystallinity, and thermal stability, of the MNPs were determined. Furthermore, the efficiency of the prepared MNPs at collecting oil spills was also investigated.

## 2. Materials and Methods

### 2.1. Materials

Sodium alginate (S.ALG), dodecylamine (DA), octadecylamine (OA), concentrated sulfuric acid, hydrochloric acid, methanol absolute, ethanol absolute, dimethylformamide (DMF), hydrated ferric chloride (FeCl_3_∙6H_2_O), hydrated ferrous chloride (FeCl_2_.4H_2_O), and ammonium hydroxide (NH_4_OH) were supplied by Sigma-Aldrich Co (Missouri, MO, USA). and were used without further purification.

Heavy crude oil was supplied by Aramco Co., Riyadh, Saudi Arabia with API and contents (wt%) of SARA 20.8°, 16.3, 25.3, 48.1, and 8.3, respectively. The specifications of the used crude oil were reported in our earlier work [20]. Brine solution (35,000 ppm) was prepared in our lab using sodium chloride salt.

### 2.2. Preparation of H.ALG

First, 5 g of S.ALG was dissolved in 250 mL of distilled water and then the dissolved salt was treated with an excess amount of hydrochloric acid (2 M). The produced precipitated alginic acid (H.ALG) was filtered, and then washed several times with distilled water, followed by the evaporation of the remaining water under reduced pressure.

### 2.3. Preparation of Methyl Alginate

In this step, H.ALG (1 g) was suspended in 200 mL methanol containing 1 mL of concentrated sulfuric acid (1 M) and was stirred for 72 h at 60 °C [21]. The solid product obtained was filtered and washed with absolute ethanol and acetone, and was finally dried in air.

### 2.4. Amidation of Methyl Alginate

About 5 g of either OA or DA was dissolved in 50 mL of DMF at 55 °C, followed by the addition of suspended methyl alginate (1 g) and the formed mixture was stirred at the same temperature for 72 h. The produced amides, OA-ALG and DA-ALG, were filtered and washed with acidified ethanol (7:1 ethanol:4 M HCl) then absolute ethanol, followed by acetone, and were finally dried in air [22]. The produced amides from the amidation reaction of methyl alginate with OA and DA were assigned as OA-ALG and DA-ALG, respectively.

### 2.5. Synthesis of MNPs 

For the synthesis of OA-MNPs and DA-MNPs, solutions of FeCl_3_∙6H_2_O and FeCl_2_∙6H_2_O (2:1 molar ratio, 10.8:3.9 g dissolved in 200 mL of distilled water) were mixed with either of OA-ALG or DA-ALG solution (4 g dissolved in 200 mL of isopropanol). The mixtures were heated up to 50 °C under nitrogen atmosphere and then NH_4_OH solution (28%, 25 mL) was added dropwise using a dropping funnel with continuous vigorous stirring. The mixtures were stirred for an extra 1 h to ensure completeness of the reaction and, finally, the produced OA-MNPs and DA-MNPs were easily separated using an external magnet, the product was washed several times with isopropanol followed by water, and was finally dried at ambient temperature. 

### 2.6. Characterization of MNPs 

The chemical structures of the as-synthesized amides and the MNPs capped with OA-ALG or DA-ALG were elucidated using Fourier-transform infrared spectroscopy (FTIR) (Nicolet 6700 spectrometer, Thermo Fisher Scientific Co., Waltham, MA, United States) and nuclear magnetic resonance (NMR) spectroscopy (Avance DRX-400 spectrometer, Bruker Co., Billerica, MA, United States). The crystal lattice structures of OA-MNPs and DA-MNPs were analyzed using powdered X-ray diffraction (XRD) (BDX-3300 diffractometer, Beijing University Equipment Manufacturer, Beijing, China). The thermal stability of OA-MNPs and DA-MNPs was evaluated using thermogravimetric analysis (TGA) (DSC-60, Shimadzu Co., Kyoto, Japan). The particle size (PS) and polydispersity index (PDI) of OA-MNPs and DA-MNPs were determined using dynamic light scattering (DLS) (Malvern Instrument Co., Malvern, United Kingdom). The morphology and structure of OA-MNPs and DA-MNPs was observed using a transmission electron microscope (TEM) (JEM-2100F, Jeol Co., Tokyo, Japan) and the magnetic properties were evaluated using a vibrating sample magnetometer (VSM) (LDJ-9600 Electronics, Tory, MI, United States). The statistical analysis was carried out using one-way analysis of variance (ANOVA) with significance set as *p* < 0.01 and a highly significant value of *p* < 0.005.

For measuring the contact angle of brine droplet at the surface of OA-MNPs and DA-MNPs, a suitable amount of OA-MNPs or DA-MNPs was dispersed in chloroform, spread onto the surface of glass slide, and dried at 50 °C in an oven. These steps were repeated several times until we saw the formation of a thin film of OA-MNPs or DA-MNPs at the surface of the glass. For DLS measurements, MNPs was sonicated in ethanol (1 mg/mL), and 2 mL of dispersed MNPs was injected into a glass cuvette and put in the sample holder for measuring.

### 2.7. Efficacy of MNPs as Oil Spill Collectors

Heavy crude oil (2 mL) was injected over the surface of brine solution (400 mL) in a 1000 mL beaker. Different ratios of OA-MNPs or DA-MNPs related to the contents of crude oil were spread as powder to the oil and were blended for 1 min using a glass rod. The mixture was left for 5 min and the oil on the surface of the brine was collected using an external magnet (a permanent Nd-Fe-B magnet 4300 Gauss). The collected oil over MNPs was washed in a beaker using chloroform, followed by the evaporation of chloroform under reduced pressure using a rotary evaporator (Büchi Co, Flawil, Switzerland). The amount of collected crude oil was determined, and the collections efficiency of OA-MNPs, or DA-MNPs was evaluated using the following equation:

(1) $$E\left(\%\right)=\left(\frac{V_{o}}{V_{1}}\times 100\right)$$

where V_o_, V_1_ are the volume of collected and original crude oil, respectively.

For measuring the reusability of OA-MNPs and DA-MNPs, the used MNPs were recollected after finishing the accumulation of crude oil, washed several times with chloroform and then with ethanol. Finally, the MNPs were air-dried before using them in a new cycle of crude oil removal test.

## 3. Results and Discussion

The present study aimed to use a new capping agent derived from S.ALG for the synthesis of hydrophobic MNPs for the efficient collection of oil spills, as S.ALG is widely available and is a naturally occurring polymer. For the study, the S.ALG was first converted into the corresponding acid, H.ALG, and was then esterified using methanol. The produced methyl ester was reacted with DA and OA to form the corresponding amides, OA-ALG, and DA-ALG, respectively. The OA-ALG and DA-ALG were successfully used as capping agents for MNPs to produce corresponding OA-MNPs and DA-MNPs, respectively. These amides were applied as capping agents due to their strong ability to interact with crude oil components via different interactions such as hydrogen bonds, Van deer Waals, and polarity induction forces. Therefore, we hypothesize that the use of the as-prepared amides as capping agents for MNPs will enhance their dispersity in crude oil rather than brine, and increase their efficacy for the collection of crude oil from the surface of brine.

**Figure 1:**
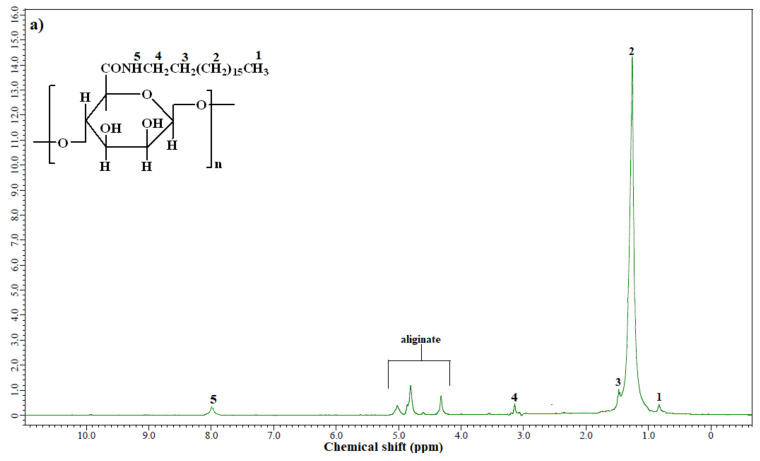

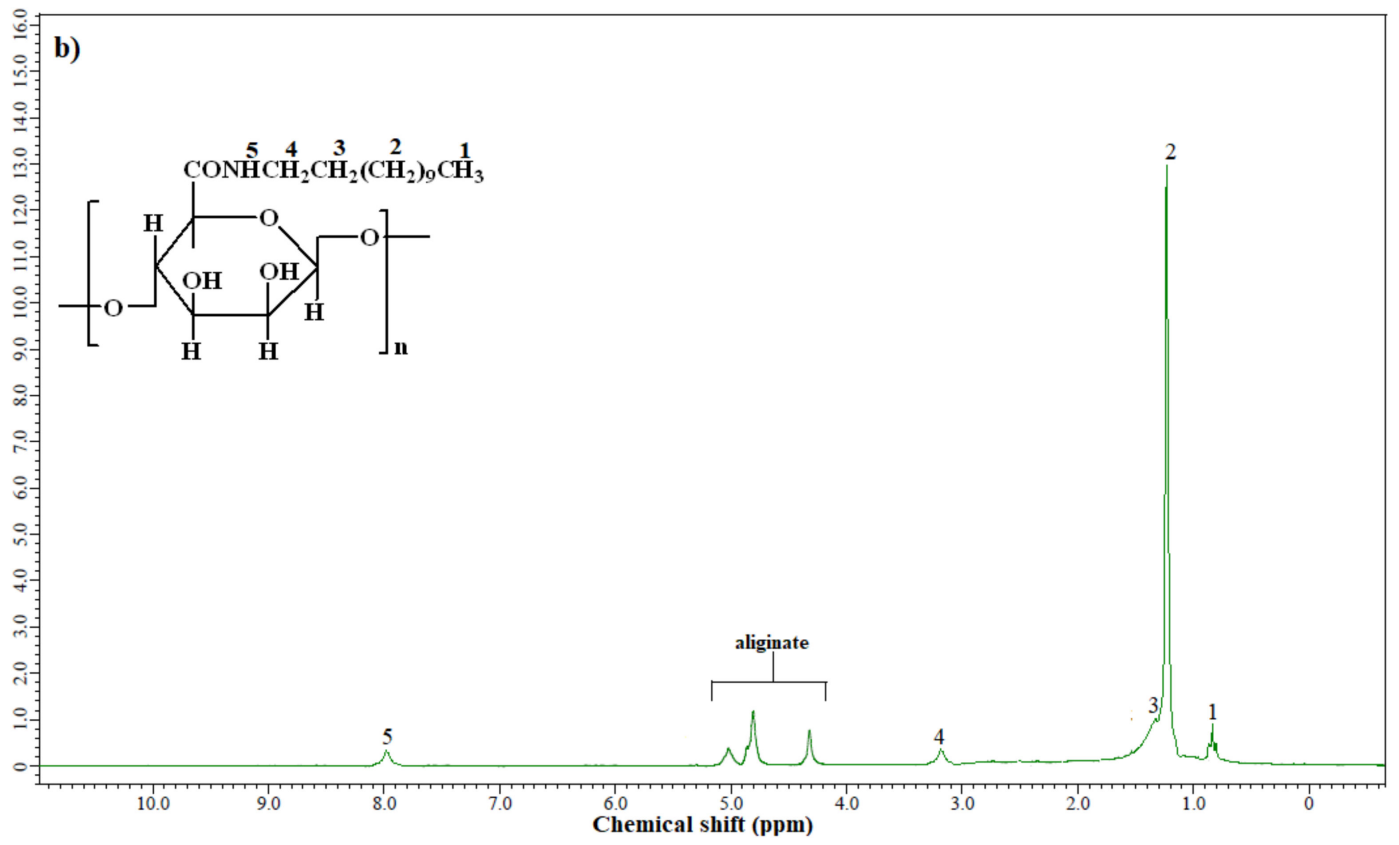
^1^H-NMR spectroscopies of (**a**) OA-ALG and (**b**) DA-ALG.

The chemical structures of OA-ALG and DA-ALG were elucidated using ^1^H-NMR spectroscopy as presented in Figure 1a,b.

**Figure 2:**
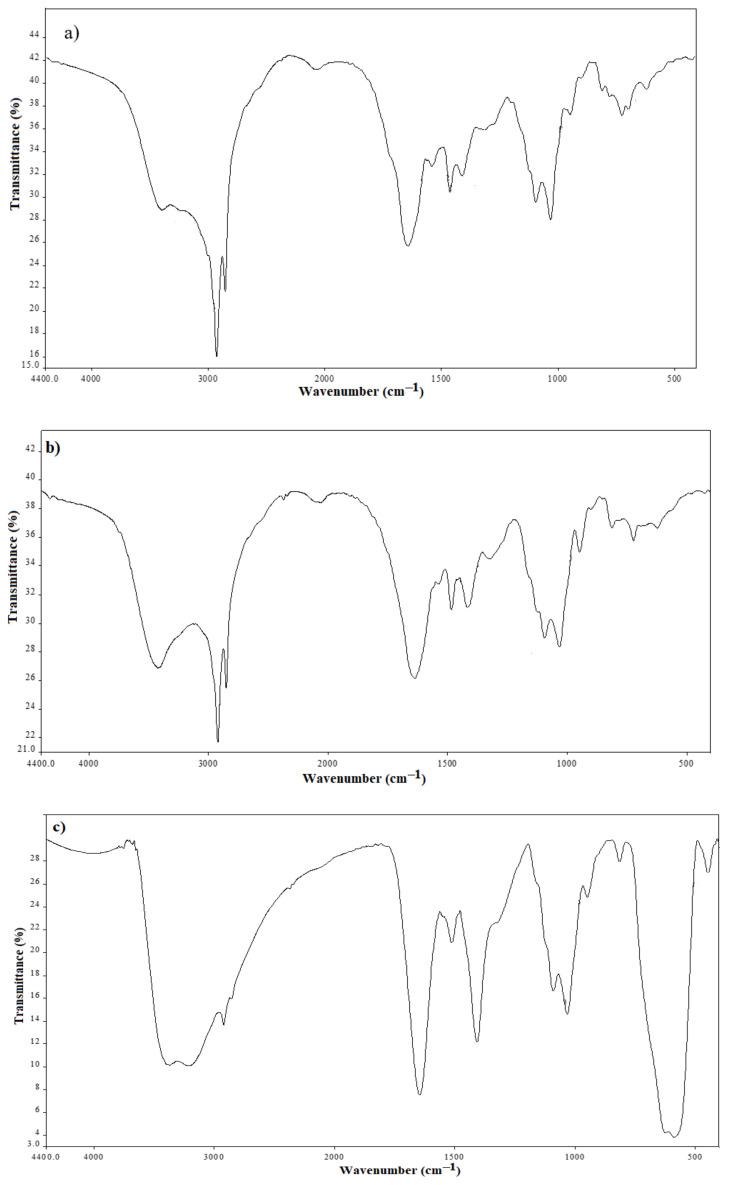

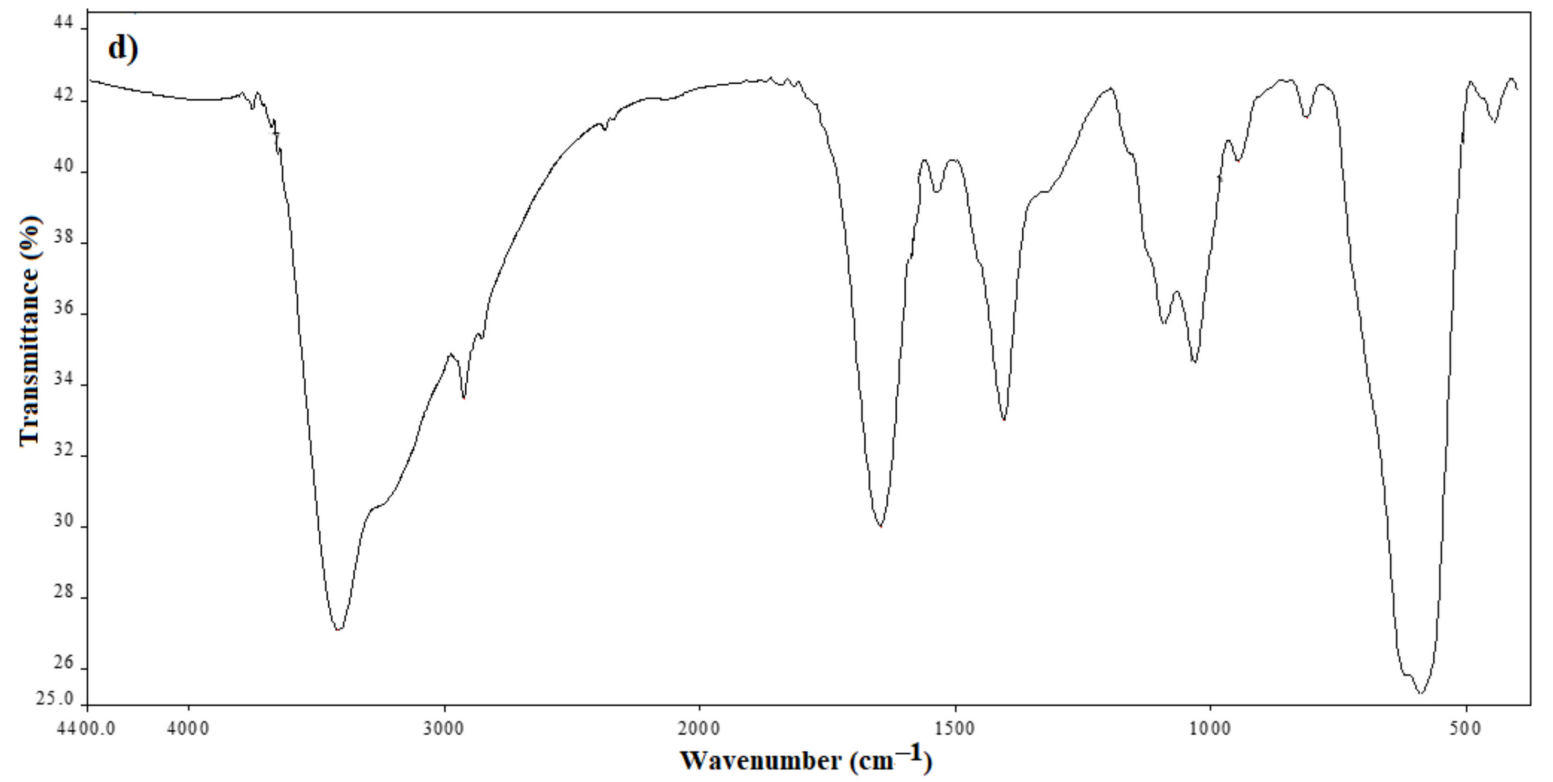
FTIR spectra of (**a**) DA-ALG, (**b**) OA-ALG, (**c**) OA-MNPs, and (**d**) DA-MNPs.

As observed from Figure 1a,b, the peaks of alkyl chain protons of OA and DA appeared at 0.88 ppm, 1.21 ppm, 1.3 ppm, and 3.18 ppm. The proton of amide (–NH) resonated at 8.2 ppm and, additionally, alginate protons appeared at between 4.3 and 5.17 ppm [23], indicating that the amidation reaction occurred successfully. The active functional groups in the as-synthesized amides and MNPs were also investigated using FTIR as presented in Figure 2a,b. From the FTIR spectra of OA-ALG and DA-ALG (Figure 2a,b), the stretching vibrations of hydroxyl groups appeared as broad bands centered at 3399 cm^−1^ and 3426 cm^−1^ for OA-ALG and DA-ALG, respectively. The stretching and binding bands of saturated alkyl chains appeared at around 2920 cm^−1^, 2850 cm^−1^, and 1464 cm^−1^ for both amides. The stretching absorption band of the carbonyl group was observed at around 1655 cm^−1^ for both amides [23]. The vibration bands in the range 1100–1000 cm^−1^ were assigned to the glyosidic bonds (O–C–O) in the alginate [24]. In a similar way, in the FTIR spectra of OA-MNPs and DA-MNPs (Figure 2c,d), the same bands were noticed indicating the interaction of the functional groups of these amides with those of MNPs. Additionally, the appearance of new bands at around 588 cm^−1^ (Fe–O) indicated the formation of pure MNPs without other iron oxides. Notably, an increase in the intensity of this band indicates an increased ratio of MNPs in OA-MNPs and DA-MNPs samples.

**Figure 3:**
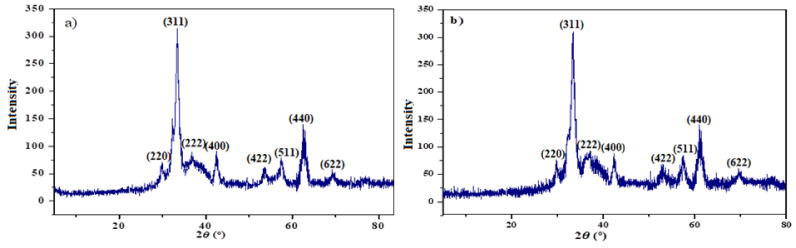
XRD-diffraction pattern of (**a**) OA-MNPs and (**b**) DA-MNPs.

The iron oxide type, beside the crystalline structure, was also investigated by the powdered XRD as presented in Figure 3a,b. The XRD of the tested samples exhibited characteristic reflection patterns at 2θ = 30°, 35°, 37°, 43°, 54°, 57°, 63° and 71°, which correspond to (220), (311), (222), (400), (422), (511), (440), and (622), respectively. The diffraction patterns were matched well with the standard magnetite XRD pattern (JCPDS file No: 00-003-0863), which informs us that the crystal structure of MNPs is not affected by the modification of their surfaces with OA-ALG or DA-ALG.

**Figure 4:**
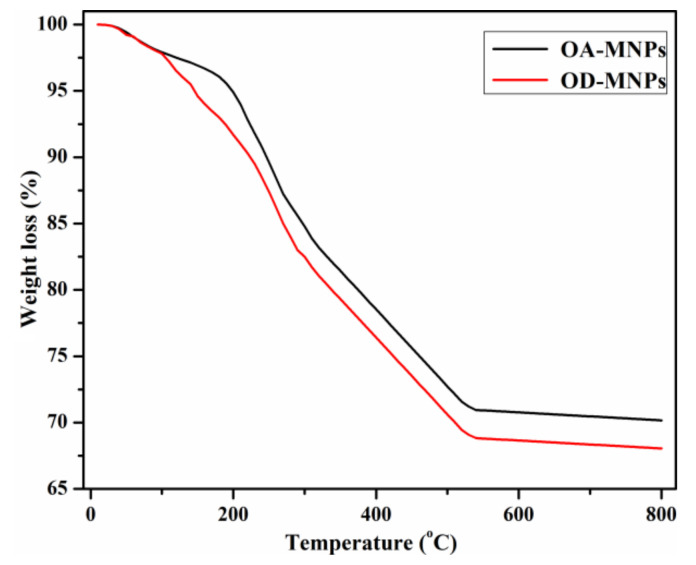
TGA thermograms of OA-MNPs and DA-MNPs.

The thermal stability of OA-MNPs and DA-MNPs was evaluated using TGA as presented in Figure 4. The data showed that the main decomposition occurred between 100 and 540 °C. The initial weight loss with an increase of temperature up to 200 °C is ascribed to the removal of physisorbed water and other solvents during the synthesis and purification steps of MNPs. However, the degradation in the region 200–540 °C could be ascribed to the decomposition of DA-ALG, OA-ALG constituents. Additionally, the data also exhibited a larger increase in the magnetite content of DA-MNPs (70.17%) than that of OA-MNPs (68.05%).

**Figure 5:**
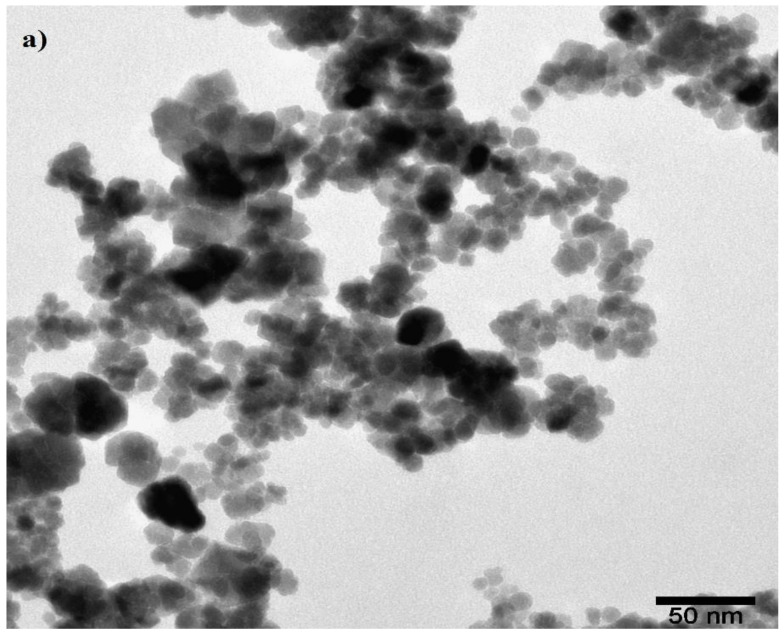

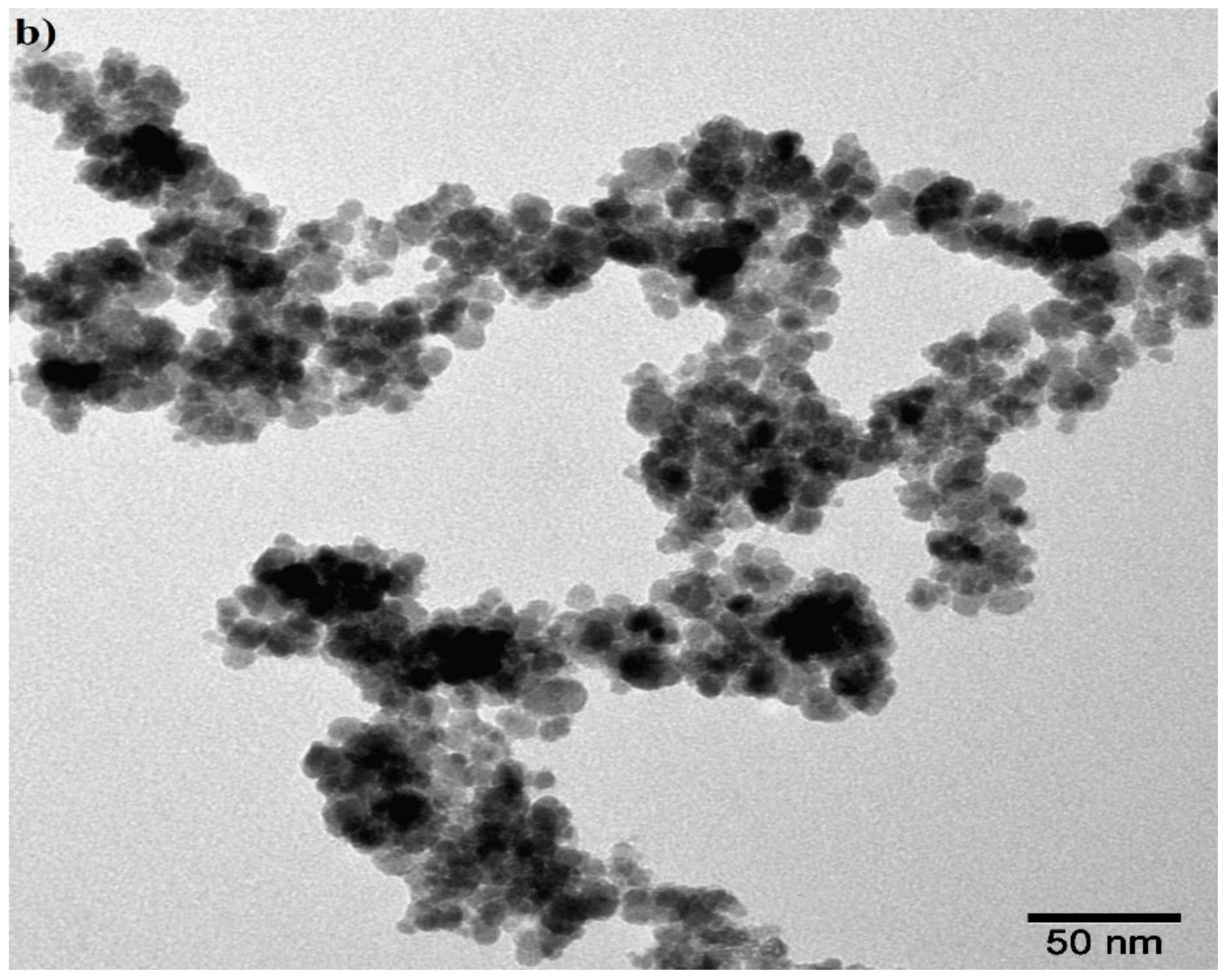
TEM of (**a**) OA-MNPs and (**b**) DA-MNPs.

**Figure 6:**
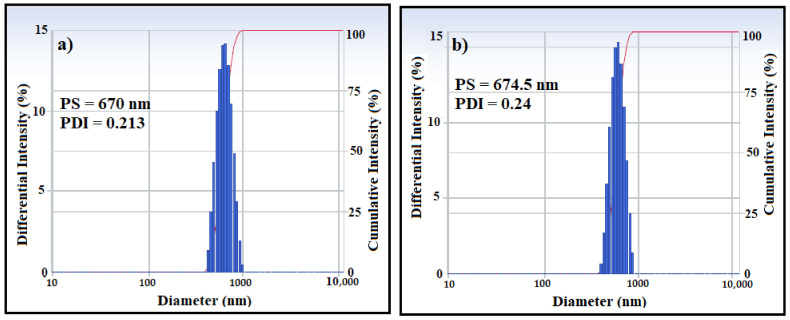
Particle size distribution of (**a**) OA-MNPs and (**b**) DA-MNPs.

The morphology and structure of OA-MNPs and DA-MNPs were observed using TEM micrographs, which are presented in Figure 5. From the images, the micrographs exhibited a limited variation in the particle size with irregular structures with an average diameter of 9.5 ± 3 nm. Notably, OA-MNPs and DA-MNPs appeared in cluster form due to the magnetic nature of MNPs [25]. The tendency of OA-MNPs and DA-MNPs to form clusters was also indicated by DLS measurements as presented in Figure 6a,b. The PS and PDI were found to be 670 nm and 0.213 respectively for OA-MNPs, while they were 674.5 nm and 0.24 for the DA-MNPs, respectively. The difference between PS measured by TEM and DLS reflected the agglomeration status of these MNPs in ethanol. 

**Figure 7:**
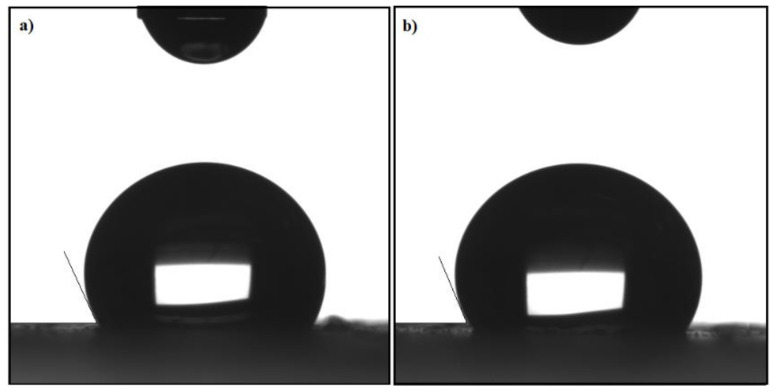
Contact angles of (**a**) DA-MNPs and (**b**) OA-MNPs.

As was reported earlier, the efficacy of MNPs in the collection of an oil spill is affected by their dispersion or precipitation in either crude oil or brine [4]. This efficacy increases as the disparity of these nanoparticles in crude oil is increased. For that, the disparity of as-synthesized MNPs in low polar and brine was tested. They exhibited high disparity in xylene, toluene, chloroform, and other low polar solvents, while there was no easy dispersion in brine. In addition, the hydrophobicity of OA-MNPs and DA-MNPs was tested using contact angle measurements as was reported in the Experimental section and presented in Figure 7. The contact angles of brine drops on the OA-MNP and OA-MNPs surfaces are 116° and 110°, respectively.

**Figure 8:**
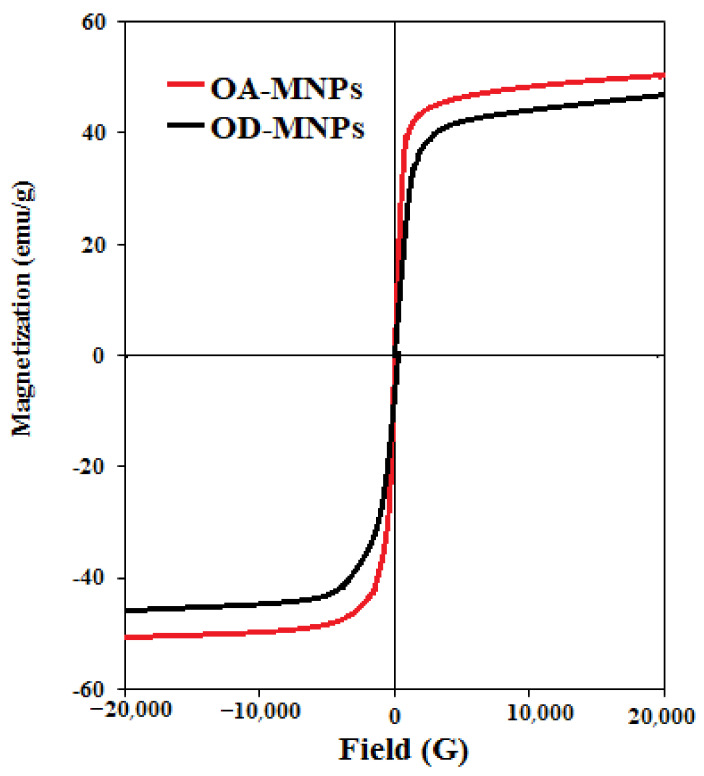
VSM magnetization curves of OA-MNPs and DA-MNPs.

Notably, these results reflected the hydrophobicity of OA-MNPs and DA-MNPs. Furthermore, the OA-MNPs showed higher hydrophobicity than DA-MNPs, which may be ascribed to an increase in its hydrophobicity due to the presence of a longer alkyl chain in the composition of OA-MNPs’ capping agent. In addition to the importance of MNPs’ hydrophobicity for the collection of oil spills, the response of the as-synthesized MNPs to an external magnetic field also represents one of the important parameters for enhancing their efficacy. The magnetic properties of OA-MNPs and DA-MNPs were evaluated at room temperature and are represented in Figure 8. The magnetization values of OA-MNPs and DA-MNPs are 52.4 and 47.2 emu/g, respectively. These values confirm the ability of the as-synthesized MNPs to collect OA-MNPs and DA-MNPs using an external magnetic field. Additionally, an increase of the magnetization value of OA-MNPs reflects a higher magnetic content (lower capping agents) than that of DA-MNPs. The TGA results analysis (provided in Figure 4) also confirmed these observations. Furthermore, the magnetization curve indicates the low values of coercive force and magnetic remanence, which suggests the absence of remanence or coercivity of the formed MNPs.

The hydrophobicity and the ability of OA-MNP and DA-MNPs to respond to the external magnetic field enhance their chance to serve as oil spill collectors. Their efficacy at collecting oil spills at different MNPs:crude oil ratios (1:1 to 1:50) was tested and is listed in Table 1. The results showed an increased efficacy of MNPs as their ratios to crude oil increased. Notably, the OA-MNPs exhibited a higher collection efficacy than that of DA-MNPs and such an increase may be attributed to an increase in the hydrophobicity of OA-MNPs because of the availability of longer alkyl chains (C_18_) in the structure of the capping agent, enhancing its interaction with crude oil components. From the economic point of view, the ratio 1:10 is the best ratio due to the ability of OA-MNPs and DA-MNPs to collect 93% and 87% of an oil spill, respectively.

**Figure 9:**
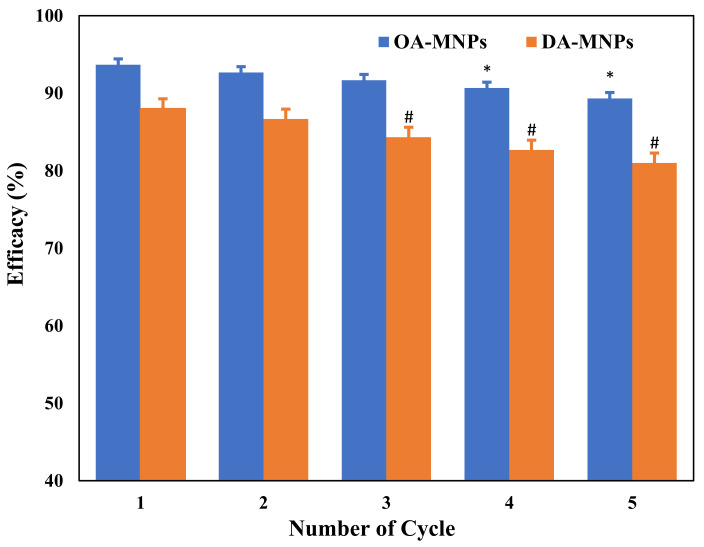
Reusability comparison of OA-MNPs and DA-MNPs. All data represented as mean ±, SD; * *p* < 0.05 against first cycle value; # *p* < 0.05 against first cycle value.

The reusability of OA-MNPs and DA-MNPs was tested in five cycles and the results are presented in Figure 9. A ratio of 1:10 (MNPs:heavy crude oil) was selected for evaluating the efficacy of recovered MNPs for the collection of an oil spill. The data indicate that the efficacy of MNPs is reduced slightly with an increasing number of cycles and this is perhaps linked to the alteration occurring in the polarity of MNPs. We observed from the performance analysis that there was only a 4% reduction (93% to 89%) [4].

## 4. Conclusions

To conclude, the present study deals with the synthesis of two amides—OA-ALG and DA-ALG—followed by their applicability as a capping agent for the preparation of MNPs, OA-MNPs and DA-MNPs, respectively. The formed OA-MNPs and DA-MNPs were analyzed for their physicochemical properties along with testing on their collection of oil spills. From the characterization analysis, the incorporation of MNPs with OA-ALG and DA-ALG was confirmed by the FTIR and powdered XRD analyses. The contact angle measurements confirmed the hydrophobicity of OA-ALG and DA-ALG, which suggests their higher ability to disperse in crude oil as compared to brine. The TGA analysis revealed an increased magnetite content of DA-MNPs compared to OA-MNPs. The nanostructures of OA-MNPs and DA-MNPs were elucidated from the TEM micrographs where the MNPs were noticed to be irregular with an average diameter of 9.5 ± 3 nm. In DLS measurements, an increase in the average diameter of OA-MNPs and DA-MNPs was found to be 670 and 674.5 nm, reflecting their magnetic nature in ethanol where they tend to form aggregates. The magnetization values of OA-MNPs and DA-MNPs suggested their ability to respond to an external magnetic field. Additionally, the OA-MNPs and DA-MNPs exhibited high efficacy in serving as oil spill collectors even when using low ratios of MNPs. The studies indicated that the OA-MNPs have higher efficacy than do DA-MNPs. This may be ascribed to an increase in their hydrophobic behavior due to the presence of longer alkyl chains in the structure of the capping agent, which in other words enhances the interaction of OA-MNPs with crude oil components. Further, the reusability of OA-MNPs and DA-MNPs was confirmed for five cycles, showing a slight loss in their efficacy with every repeating cycle. Finally, the wide availability and green character of S.ALG for the preparation of amides and the ability to apply them as capping agents for MNPs makes this approach more acceptable for oil spill remediation and collection purposes.

## Figures and Tables

**Table 1:** Efficacy (%) of OA-MNPs and DA-MNPs in the collection of an oil spill.

MNPs	Efficacy (%) Using Different (MNPs:Crude Oil) (Wt.:Vol%)			
	1:1	1:10	1:25	1:50
OA-MNPs	96	93	87	78
DA-MNPs	90	87	75	67

## Data Availability

The data presented in this study are available on request from the corresponding author.
